# Evaluation of the relationship between left atrial strain and exercise tolerance in patients with hypertrophic cardiomyopathy by treadmill stress echocardiography

**DOI:** 10.3389/fcvm.2023.1168514

**Published:** 2023-05-15

**Authors:** Ye Su, Chunmei Li, Lixue Yin

**Affiliations:** ^1^ School of Medicine, University of Electronic Science and Technology of China, Chengdu, China; ^2^ Department of Cardiovascular Ultrasound, Sichuan Provincial People’s Hospital, University of Electronic Science and Technology of China, Chinese Academy of Sciences Sichuan Translational Medicine Research Hospital, Chengdu, China; ^3^ Ultrasound in Cardiac Electrophysiology and Biomechanics Key Laboratory of Sichuan Province, Sichuan Provincial People's Hospital, University of Electronic Science and Technology of China, Chengdu, China

**Keywords:** HCM, METS, left atrial reservoir strain, left atrial conduit strain, left atrial contraction strain

## Abstract

**Objective:**

The aim of this study is to evaluate the left atrial strain (LAS) in patients with hypertrophic cardiomyopathy (HCM) by treadmill exercise stress echocardiography, combined with three-dimensional speckle tracking technology, for predicting exercise tolerance.

**Methods:**

A total of 97 patients with HCM who underwent treadmill exercise stress echocardiography were recruited in Sichuan Provincial People's Hospital between January 2018 and January 2021, and 30 control subjects were selected to be included in the normal group. HCM patients with their metabolic equivalents (METS) ≤ 6.0 were included in the HCM-1 group, while those with METS > 6.0 were included in the HCM-2 group. The LAS and exercise tolerance were analyzed. The ultrasound parameters that could predict a decrease in exercise tolerance were screened, and a predictive model was constructed.

**Results:**

It was found that METS, Rest-LASr, Rest-LAScd, and Rest-LASct were significantly lower in HCM patients than those in normal controls. There was a significant difference in age, Target_HR, LVMI, LAVI, *E*/*e*'-Rest, *E*/*e*'-Peak, Rest-LASr, Rest-LAScd, and Rest-LASct between the HCM-1 and the HCM-2 groups. LASr is an independent resting echocardiographic predictor of METS ≤ 6.0. LASr remained significant for predicting different subtypes (AHCM, asymmetric HCM, and obstructive HCM). Rest-LASr (AUC 0.990) was better at predicting METS ≤ 6.0 than Peak-*E*/*e*' (AUC 0.753). A multivariate model (LASr + Age + Target_HR) was established for METS prediction.

**Conclusion:**

Left atrial reservoir strain (LASr) has the strongest association with METS ≤ 6.0. The LASr is an independent resting predictor of METS ≤ 6.0 and has a good performance record in predicting different subtypes of HCM. Compared with the traditional parameters, Peak-*E*/*e*' and Rest-*E*/*e*', Rest-LASr is the best predictor. Rest-LASr can serve as a reliable method for HCM patients who are unable to undergo exercise testing but require an urgent evaluation of their METS, which provides a basis for clinical treatment decision-making and treatment effect evaluation.

## 1. Introduction

Hypertrophic cardiomyopathy (HCM) is an autosomal inherited cardiomyopathy characterized by left ventricular hypertrophy, and is mainly caused by mutations in the sarcomere protein genes (MYH7 and MYBPC3 are the most common pathogenic genes) (1). The presence of multiple types of anatomic and functional abnormalities and the broad heterogeneity in the clinical manifestations in HCM make risk stratification and accurate clinical treatment decision challenging. The 2020 European Society of Cardiology (ESC) guidelines (2) emphasize the multiple benefits of regular exercise but stipulates an individualized risk assessment and exercise program that can reconcile the balance between exercise and risk. Metabolic equivalents (METS), which can reflect exercise tolerance, have been demonstrated to be a strong independent predictor of cardiovascular disease risk and poor prognosis, and higher METS were associated with lower mortality (3). Currently, stress echocardiography is the first-line imaging technique that can be used to detect METS and cardiac function in clinical practice; however, there are many HCM patients who are unable to undergo stress tests for various reasons (such as severe outflow tract obstruction, physical disability, etc.) in clinical practice. Therefore, a parameter that can reflect METS without exercise testing would be better suited to clinical needs. Three-dimensional speckle tracking technology (3D-STI), a new technique of echocardiography discovered in recent years, allows a quantitative evaluation of the mechanical function of the atria by analyzing atrial myocardial deformation and the working characteristics of the atrial myocardium, and has been shown to have predictive value for the risk of adverse cardiovascular events. Wazzan et al. (4) found that impaired peak left atrial strain (LAS) in patients with HCM was associated with the risk of stroke. Zegkos et al. (5) found that left atrial reservoir strain (LASr) predicted ventilation efficiency in patients with HCM. However, it is unclear whether left atrial strain can be used as a parameter to reflect METS in patients with HCM. Therefore, this study hypothesized that reduced left atrial strain could predict impaired METS in HCM patients. This study used 3D-STI and stress echocardiography to assess the relationship between LAS and METS and to further verify that left atrial strain at the resting stage could predict METS in HCM patients.

## 2. Method

### 2.1. Study population

A total of 120 adult patients who were diagnosed clinically with HCM in Sichuan Provincial People’s Hospital between January 2018 and January 2021 were included in this prospective study, and 23 patients with incomplete data or poor images were excluded. The study ultimately included 97 patients. There were 68 males and 29 females in the HCM group, with an average age of 46 ± 13 years. In approximately 40–60% of patients, genetic mutations were found to be cause for HCM, while in the remaining percentage of patients, the underlying causes remained unclear (1). The clinical diagnostic criteria of HCM were devised according to the 2017 Chinese Guidelines for the Diagnosis and Treatment of Hypertrophic Cardiomyopathy in Adults (6) and the 2014 ESC Guidelines (7) and the 2020 AHA/ACC Guidelines (8). The clinical definition of HCM is a maximal end-diastolic wall thickness of ≥15 mm in one or more left ventricle (LV) myocardial segment detected by echocardiography or cardiovascular magnetic resonance in the absence of another cardiac, systemic, or metabolic disease capable of producing LV hypertrophy (LVH). The diagnostic criteria for apical hypertrophic cardiomyopathy (AHCM) are LVH predominating in the LV apex, with a wall thickness of ≥15 mm in the apex and a maximal apical-to-posterior wall thickness ratio of ≥1.5, with a “spadelike” configuration of the LV cavity in the end diastole and “giant” negative precordial T-waves on electrocardiography. The diagnosis of asymmetrical HCM is based on the presence of significant LVH and a septal-to-posterior wall thickness ratio of >1.3. Hypertrophic cardiomyopathy is divided into non-obstructive and obstructive types according to hemodynamics; non-obstructive type is defined as an instantaneous peak Doppler LV outflow tract pressure gradient (LVOT-PG) < 30 mmHg at rest, while obstructive HCM is defined as LVOT-PG ≥30 mmHg at rest. The exclusion criteria for the HCM group patients were as follows: hypertension, coronary heart disease, moderate or high aortic valve stenosis or other obvious symptoms causing myocardial hypertrophy, respiratory system diseases, and other diseases affecting cardiac function; other contraindications related to treadmill stress echocardiography (9); and poor image quality. In addition, 30 healthy subjects who were matched with those in the HCM group were selected to be included as the normal group for undergoing treadmill exercise stress echocardiography, and they constituted 16 males and 14 females, with an average age of 46 ± 6 years. The electrocardiogram of all HCM patients showed a sinus rhythm without a conduction system block. This study was approved by the Ethics Committee of Sichuan Provincial People’s Hospital and was performed in accordance with the Declaration of Helsinki (as revised in 2013). All patients signed the informed consent form for the treadmill exercise stress test. The study procedures strictly followed the rules for the protection of patient privacy, and all data were anonymized.

### 2.2. Equipment and methods

#### 2.2.1. Electrocardiogram of treadmill exercise

The SunTechTango synchronized ambulatory blood instrument (SunTech Medical Instruments, NC, USA) and the MortaraX-Scribe treadmill motion analysis system (Mortara Instrument, Milwaukee, WI, USA) were used to perform symptom-limited treadmill exercise cardiography. All subjects were tested by using the BRUCE protocol. Synchronized 12-lead electrocardiogram and blood pressure were measured during rest and exercise. All subjects discontinued the use of receptor blockers or calcium channel blockers for at least 24 h prior to the start of the trial. Resting systolic and diastolic blood pressure were measured and synchronized 12-lead electrocardiograms were recorded simultaneously. Exercise termination metrics were updated according to the 2002 ACC/AHA exercise testing guidelines (10): ① ST-segment elevation of >1.0 mm in no pathological Q-wave lead (except V1 or aVR); ② systolic blood pressure decreased by >10 mmHg with other evidence of ischemia (such as the ST segment is horizontally or diagonally depressed more than 0.1mv (except the AVR), or the T wave is towering, positive and negative in both directions, inverted, etc.); ③ moderate to severe angina pectoris; ④ CNS symptoms such as ataxia, dizziness, and syncope; ⑤ signs of hypoperfusion such as cyanosis and pallor; ⑥ persistent ventricular tachycardia; and ⑦ technical difficulties in checking electrocardiogram or systolic blood pressure. Snader et al. (11) conducted a single-center study involving 3,400 patients on exercise tolerance-METS for predicting all-cause mortality. When using METS ≤ 6.0 as the cutoff value to assess impaired exercise tolerance, the effect on all-cause mortality was significant, and the predictive value was the highest. Thus, we selected METS ≤ 6.0 as the critical value. Specifically, HCM patients with METS ≤6.0 and >6.0 were included in the HCM-1 and HCM-2 groups, respectively.

The GE Vivid E95 color Doppler ultrasound diagnostic apparatus with 4V-D probe (frequency = 1.5–4.0 MHz; GE Medical Systems, Milwaukee, WI, USA) was used. All subjects were positioned in the left lateral position and their electrocardiogram was synchronously recorded. Resting systolic and diastolic blood pressure measurements were taken by two experienced sonographers. Physicians performed echocardiography and continuously acquired dynamic images of the apex for at least five cardiac cycles (image frame rate ≥60 frames/s). The obtained images were digitally stored and analyzed offline using the EchoPAC (203) workstation. All parameters were measured and analyzed in accordance with the American Society of Echocardiography (ASE) guidelines (12–14): Left ventricular ejection fraction (LVEF) (measured using the Simpson method), maximum left ventricular septal thickness (IVS), maximum left ventricular posterior wall thickness (LVPW), left atrial diameter (LA), early- (E) and late- (A) diastolic mitral anterior flow velocity and E/A ratio, peak early-diastolic mitral annular velocity (e) and end-diastolic peak mitral annular motion (a) and *E*/*e*' ratio, and metabolic equivalents of exercise (METS).

#### 2.2.2. Measurement parameters

**Figure 1:**
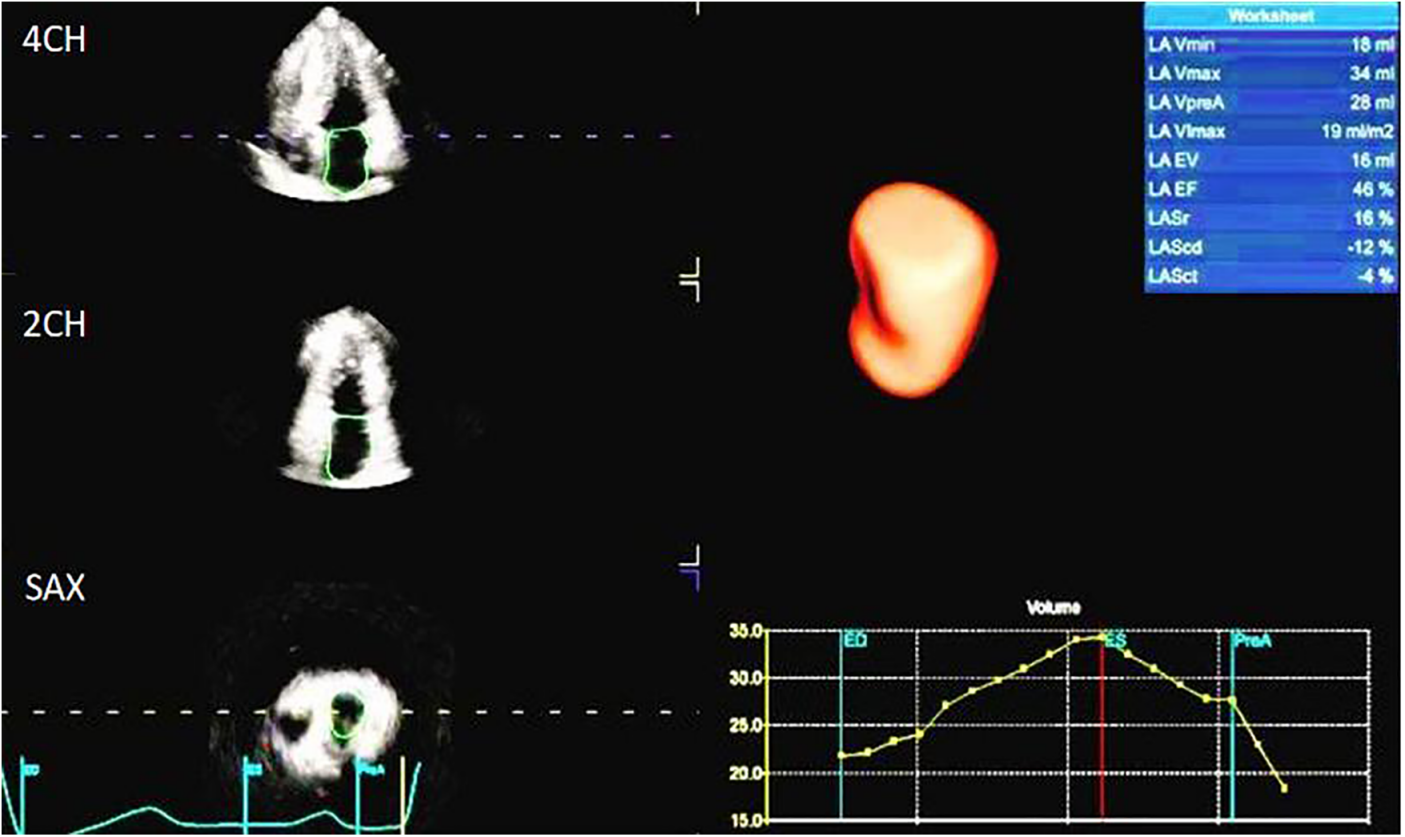
Left atrial strain in an HCM patient.

**Figure 2:**
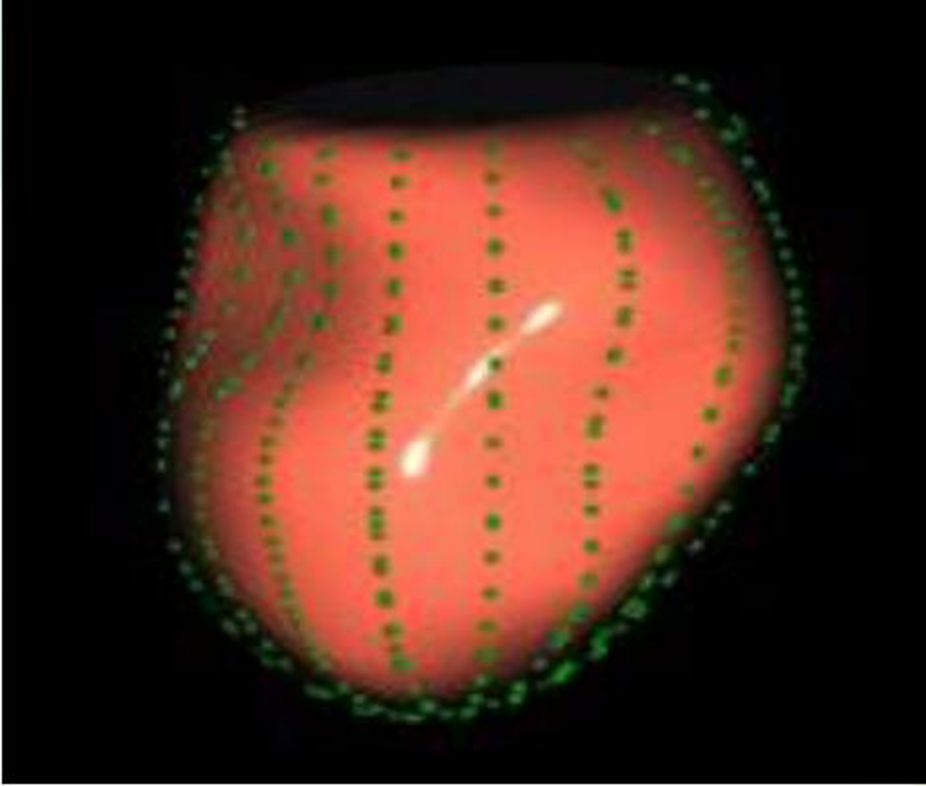
Points used to determine longitudinal lines for LA strain calculation.

The image analysis was performed using the GE-EchoPAC (203) workstation. The LAS (Figures 1, 2) was analyzed by using 3D-STI to obtain the LASr, left atrial conduit strain (LAScd), left atrial contraction strain (LASct), left atrial ejection fraction (LA-EF), left ventricular global longitudinal strain (LV-GLS), left atrial volume index (LAVI), and left ventricular mass index (LVMI). Left ventricular hypertrophy was defined as LAVI ≥34 mL/m^2^, as well as LVMI ≥125 and ≥120 g/m^2^ for men and women, respectively (15). The impaired Rest-GLS and LASr were defined as ≤ −17% and ≤ 23%, respectively, on the basis of the reference values taken from previous studies (16–18). The LASr is a measurement of LAS during systole, which occurs when the pulmonary veins flow into the left atrium at the end of the left atrium filling and just before the mitral valve opens. At this point, the left atrial wall is at the maximum stretch, resulting in a positive strain that spans from the end of diastole to the onset of ventricular filling. As the mitral valve opens, blood flows into the left ventricle from the left atrium, the left ventricle enters a rapid filling phase, and the LAS decreases until it reaches a plateau; thus, the LAScd exhibits a negative strain. LA contract strain, which is dependent on both venous return and left ventricular end-diastolic pressure (19, 20), has a negative strain value of LASct at the peak of the *P* wave; height, weight, body surface area (BSA), body mass index (BMI), systolic blood pressure (SBP), diastolic blood pressure (DBP), and heart rate (HR). All operational analyses were performed by two independent observers.

### 2.3. Statistical methods

All statistical tests were conducted with R for WINDOWS 4.0.3 software. Continuous variables that followed a normal distribution were analyzed using the *T*-test, while non-normally distributed continuous variables were determined using the Kruskal–Wallis rank-sum test. Continuous variables were expressed as mean ± standard deviation, whereas categorical variables were presented as the absolute number and relative proportion of each category. In this study, rank-sum tests were performed for “Rest-DBP,” “LVMI,” “Rest-EF,” “E/A,” “e,” “a,” “*E*/*e*'-rest,” “LAScd,” “LASct,” “LAVI,” “LA-EF,” “Peak-SBP,” “Peak-DBP,” “Peak-LVEF,” and “*E*/*e*'-Peak.” The 97 patients with BSA, AGE, LASr, LAScd, LASct, LAVI, *E*/*e*', and LAEF were analyzed by using general linear regression, in order to assess the impact of each variable on exercise capacity. The univariate that had a significant association with METS was subjected to multivariate analysis for the identification of independent METS predictors. The receiver operating characteristic (ROC) and area under the curve (AUC) were used to quantify the LASr, LAScd, LAVI, *E*/*e*-rest, and *E*/*e*-PEAK, so as to classify HCM patients with reduced exercise capacity. The DeLong test was used for comparing the AUC of LASr, LAScd, LAVI, *E*/*e*-rest, and *E*/*e*-PEAK. To evaluate the clinical value of LASr, the correlations among the clinical variables (age, Target_HR, BMI, and BSA) were determined, the corresponding *P*-value was calculated, and a multivariate linear regression model was drawn (METS∼LASr + Age + Target_HR). The variables and statistical methods involved in this study met the requirements after being reviewed by statistical experts, and the research findings were reliable.

## 3. Result

### 3.1. Patient characteristics and echocardiography

As shown in Table 1, in the HCM group, there were a total of 97 patients who all had a sinus rhythm, and their average age was (46 ± 13) years. Of these patients, 68 (70.1%) were male. During the resting stage, 55 patients (56.7%) had an increased LVMI, 77 (76.79%) had an increased LAVI (LAVI > 34 mL/m^2^), 43 (44.32%) had a Rest-*E*/*e*' ratio > 14, and 30 (30.92%) had a decreased GLS (according to the normal reference value GLS ≤ −17%). The LASr (13.22 ± 5.41%), LAScd (−7.04 ± 5.04%), and LASct (−5.20 ± 4.24%) of the HCM group were significantly reduced according to the lower limit of normal reference value. In addition, LASr < 20% was found to predict an increase in LV filling pressure and impairments (18). At the rest stage, the HR, BMI, LVMI, LAVI, *E*/*e*'_rest, *E*/*e*'-Peak, Peak_LVEF, LVPW, and IVS were all significantly higher in the HCM group than in the normal group (*P* < 0.05). At the same time, METS, Rest-*E*, Rest-*e'*, Rest-LASr, Rest-LAScd, and Rest-LASct were all significantly lower in the HCM group than in the normal group (*P* < 0.05). However, the ultrasound parameters Rest_EF and Rest_GLS, which reflect left ventricular systolic function, remained within the normal range.

**Table 1:** Clinical, echocardiographic, and exercise characteristics of HCM and normal.

	Normal group (*N* = 30)	HCM group (*N* = 97)	*P*
METS [mean (SD)]	10.46 (2.13)	9.25 (2.62)	0.023*
Gender = M (%)	16 (53.3%)	68 (70.1%)	0.14
Age (year) [mean (SD)]	46 (6)	46 (13)	0.918
Rest_HR (beat/min) [mean (SD)]	71 (8)	78 (11)	0.002*
Target_HR (beat/min) [mean (SD)]	173 (6)	172 (14)	0.748
BSA (m^2^) [mean (SD)]	6.68 (27.45)	1.69 (0.19)	0.073
BMI (kg/m^2^) [mean (SD)]	21.96 (2.22)	24.15 (3.29)	0.001*
Rest-SBP (mmHg) [mean (SD)]	121 (7)	125 (24)	0.419
Rest-DBP (mmHg) [mean (SD)]	76 (7)	77 (13)	0.934
LVPW (mm) [mean (SD)]	9 (1.11)	16.48 (5.60)	0.000*
IVS (mm) [mean (SD)]	8.17 (1.08)	12.03 (5.31)	0.000*
LVMI (g/m^2^) [mean (SD)]	115.33 (26.35)	143.53 (58.63)	0.012*
Rest_LAVI (mL/m^2^) [mean (SD)]	23.87 (2.65)	42.72 (17.43)	<0.001*
Rest-LVEF [mean (SD)]	0.66 (0.04)	0.73 (0.06)	<0.001*
Rest-E (m/s) [mean (SD)]	0.88 (0.17)	0.74 (0.23)	0.002*
Rest-A (m/s) [mean (SD)]	0.64 (0.13)	0.73 (0.27)	0.084
Rest-E/A [mean (SD)]	1.41 (0.22)	1.16 (0.56)	0.019*
Rest-e' (m/s) [mean (SD)]	0.12 (0.02)	0.06 (0.02)	<0.001*
*E*/*e*'_rest [mean (SD)]	6.05 (1.08)	14.01 (6.36)	<0.001*
Rest-LV-GLS (%) [mean (SD)]	−25.26 (2.27)	−19.68 (3.84)	<0.001*
Rest-LASr (%) [mean (SD)]	44.40 (8.20)	13.22 (5.41)	<0.001*
Rest-LAScd (%) [mean (SD)]	−27.98 (6.46)	−7.04 (5.04)	<0.001*
Rest-LASct (%) [mean (SD)]	−16.41 (3.74)	−5.20 (4.24)	<0.001*
Peak-SBP (mmHg) [mean (SD)]	167 (14)	171.03 (33)	0.57
Peak-DBP (mmHg) [mean (SD)]	77 (10)	74 (17)	0.492
*E*/*e*'-Peak [mean (SD)]	6.09 (0.83)	14.43 (6.38)	<0.001*
Peak_LVEF [mean (SD)]	0.79 (0.06)	0.83 (0.09)	0.026*

* *P* < 0.05.

### 3.2. Metabolic equivalents

As shown in Table 2, there were a total of 13 patients in the HCM-1 group with METS ≤ 6.0. Of these, five patients (38.46%) had ventricular arrhythmias, two (15.38%) had atrial arrhythmias, and five (38.46%) had tricuspid valve regurgitation grade I, 7 patients (53.84%) had tricuspid valve regurgitation grade II, while 12 (92.30%) did not have tricuspid regurgitation; 12 patients (92.30%) had mitral regurgitation grade I, 12 (92.30%) had mitral regurgitation grade II, 6 (46.15%) had mitral regurgitation grade III, and 8 (61.53%) had left ventricular outflow tract obstruction (LVOT-PG ≥ 30 mmHg) at the resting stage. A total of 84 patients in the HCM-2 group with METS > 6.0 and 5 patients (5.95%) had ventricular arrhythmias, 2 (2.38%) had atrial arrhythmias, 40 (47.61%) had tricuspid valve regurgitation class I, 11 (13.09%) had tricuspid valve regurgitation class II, 22 (26.19%) did not have tricuspid regurgitation, 52 (61.90%) had mitral regurgitation grade I, 13 (15.48%) had mitral regurgitation grade II, 2 (2.38%) had mitral regurgitation grade III, and 17 (20.24%) had LVOT- PG ≥ 30 mmHg at the resting stage. The study observed statistically significant differences between the HCM-1 group and the HCM-2 group of patients in terms of age, Target_HR, Peak-HR, Height, BSA, ventricular arrhythmias, LVMI, LAVI, Rest-A, Rest-E/A, Rest-e, *E*/*e*'-Rest, *E*/*e*'-Peak, Rest-LA-EF, Rest-LASr, Rest-LAScd, and Rest-LASct (*P* < 0.05). In the HCM-1 group, patients had a lower METS, longer medical history, and greater LVMI and LAVI. The rates of ventricular arrhythmias, mitral regurgitation, tricuspid regurgitation, and ventricular outflow tract obstruction at rest were also higher. The rates of Rest-A, *E*/*e*'-Rest, and *E*/*e*'-Peak were higher, while those of BSA, Rest-E/A, Rest-e, Rest-a, Rest-LA-EF, Rest-LASr, Rest-LAScd, and Rest-LASct were significantly lower. There were greater abnormal rates of LAVI, Rest-A, *E*/*e*'-Rest, *E*/*e*'-Peak, Rest-e, Rest-LASr, Rest-LAScd, and Rest-LASct in the HCM-1 group.

**Table 2:** Clinical, echocardiographic, and exercise characteristics based on METS in the HCM group.

	Overall	HCM-1group (METS ≤6.0)	HCM-2group (METS > 6.0)	*P*
Number	97	13	84	
Demographic and comorbidities				
Gender = M (%)	68 (70.10)	7 (53.84)	61 (72.61)	0.294
Age (year) [mean (SD)]	46.34 (13.37)	56.62 (11.30)	44.75 (13.01)	0.002*
Target_HR (beat/min) [mean (SD)]	172 (14)	158 (18)	174 (13)	<0.001*
Height (cm) [mean (SD)]	164.65 (8.26)	159.31 (9.71)	165.48 (7.75)	0.011*
Weight (kg) [mean (SD)]	65.73 (11.97)	60.77 (12.04)	66.50 (11.84)	0.109
BSA (m^2^) [mean (SD)]	1.69 (0.19)	1.60 (0.20)	1.71 (0.18)	0.049*
BMI (kg/m^2^) [mean (SD)]	24.15 (3.29)	23.79 (2.82)	24.21 (3.37)	0.673
Rest-SBP (mmHg) [mean (SD)]	125 (24)	132 (28)	124 (23)	0.294
Rest-DBP (mmHg) [mean (SD)]	77 (13)	75 (12)	77 (13)	0.711
Rest-HR (beat/min) [mean SD)]	78 (11)	81 (14)	78 (11)	0.349
Ventricular arrhythmias (%)	10 (10.30)	5 (38.46)	5 (5.95)	0.000*
Atrial arrhythmias (%)	4 (4.12)	2 (15.38)	2 (2.38)	0.149
Baseline echocardiography				
LVMI (g/m^2^) [mean (SD)]	143.53 (58.63)	199.84 (64.83)	134.81 (52.86)	<0.001*
Rest-LVEF [mean (SD)]	0.73 (0.06)	0.70 (0.09)	0.73 (0.06)	0.123
REST-LV-GLS (%) (mean SD))	−19.68 (3.84)	−18.74 (3.92)	−19.82 (3.83)	0.346
Rest-E (m/s) [mean (SD)]	0.74 (0.23)	0.80 (0.34)	0.73 (0.21)	0.287
Rest-A (m/s) [mean (SD)]	0.73 (0.27)	1.02 (0.34)	0.69 (0.23)	<0.001*
Rest-E/A [mean (SD)]	1.16 (0.56)	0.87 (0.42)	1.20 (0.57)	0.043*
Rest-e' (m/s) [mean (SD)]	0.06 (0.02)	0.04 (0.02)	0.06 (0.02)	0.01*
Rest-a (m/s) [mean (SD)]	0.08 (0.02)	0.07 (0.02)	0.09 (0.02)	0.073
*E*/*e*'-Rest [mean (SD)]	14.01 (6.36)	20.67 (10.70)	12.98 (4.72)	<0.001*
Diastolic grade				
Rest-LASr (%) [mean (SD)]	13.22 (5.41)	4.62 (1.56)	14.55 (4.49)	<0.001*
Rest-LAScd (%) [mean (SD)]	−7.04 (5.04)	−2.23 (1.17)	−7.79 (5.00)	<0.001*
Rest-LASct (%) [mean (SD)]	−5.20 (4.24)	−1.69 (3.73)	−5.74 (4.07)	0.001*
Rest-LAVI (mL/m^2^) [mean (SD)]	42.72 (17.43)	77.31 (20.94)	37.37 (8.46)	<0.001*
Rest-LA-EF (%) [mean (SD)]	37.62 (10.27)	27.00 (8.69)	39.26 (9.52)	<0.001*
Exercise parameters				
Peak-SBP (mmHg) [mean (SD)]	171 (33)	158 (40)	173 (31)	0.132
Peak-DBP (mmHg) [mean (SD)]	74 (17)	74 (13)	74 (18)	0.932
Peak-HR (beat/min) [mean (SD)]	157 (23)	124 (15)	162 (19)	<0.001*
*E*/*e*'-Peak [mean (SD)]	14.43 (6.38)	19.26 (6.95)	13.68 (5.99)	0.003*
Peak-LVEF [mean (SD)]	0.83 (0.09)	0.83 (0.08)	0.83 (0.10)	0.742

* *P* < 0.05.

### 3.3. Association of METS

**Figure 3:**
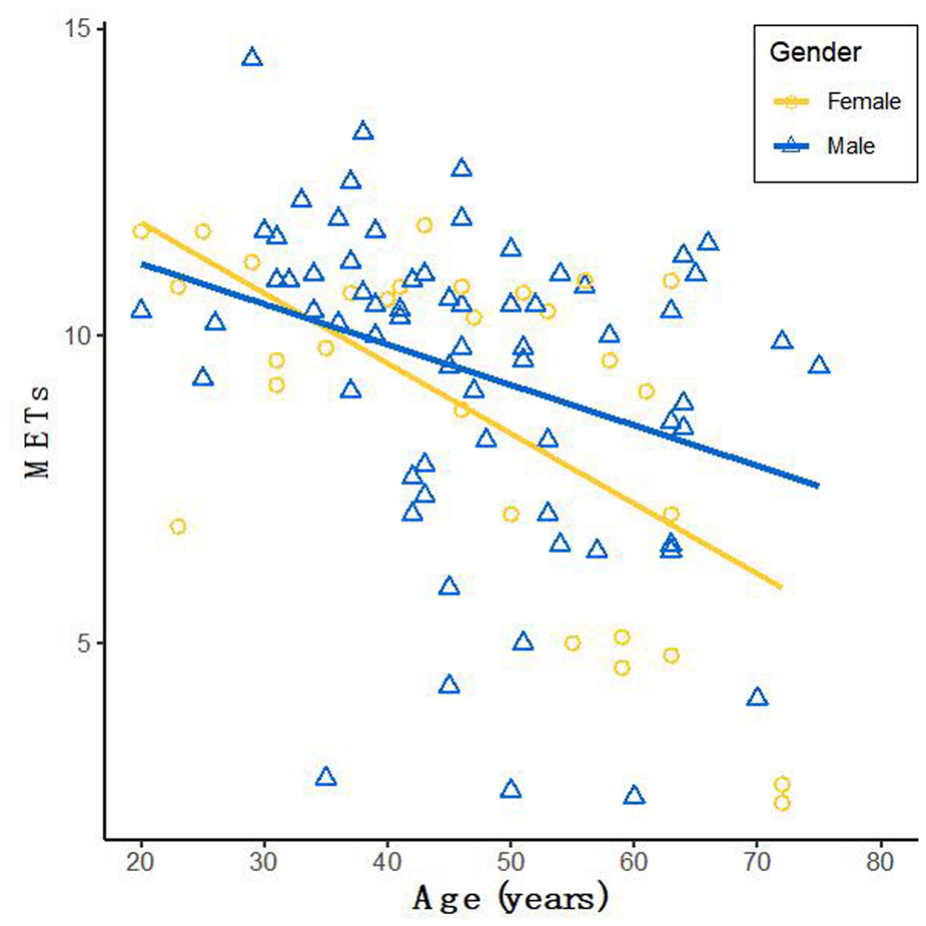
METS with increasing age in men and women.

The characteristics between the HCM-1 group and the HCM-2 group were compared using a correlation analysis, which revealed that METS had the strongest association with Rest-LASr (*r* = 0.820; *P* < 0.01), followed by LAVI (*r* = −0.662; *P* < 0.01), Rest-LAScd (*r* = −0.624; *P* < 0.01), Age (*r* = −0.495; *P* < 0.01), and Peak-*e*' (*r* = 0.466; *P* < 0.01). Males performed better than females as they grew older, as seen by METS (Figure 3). The association between METS and *E*/*e*'-Rest was the least (*r* = −0.241; *P* < 0.01), followed by BSA (r = 0.212; *P* < 0.05), A (*r* = −0.393; *P* < 0.01), Rest-*e*' (*r* = 0.297; *P* < 0.01), LA_EF (*r* = 0.391; *P* < 0.01), and LVMI (*r* = −0.210; *P* < 0.05). METS was substantially correlated with the variables Rest-LASr, Rest-LAScd, Rest-LASct, Rest-*E*/*e*', Rest-LAVI, Rest-LVMI, Rest-LA_EF, Age, BMI, BSA, and Target_HR. The contribution of each variable to METS was determined by using general linear regression (as displayed in Table 3). Age, BSA, and Target_HR were independent clinical predictors of METS (as in Model 1: Clinical in Table 3). In order to discover the best predictor of METS, we combined the analysis of Rest-LASr, Rest-LAScd, Rest-LASct with Rest-*E*/*e*', Rest-LAVI-MAX, Rest-LA_EF, and LVMI (such as Model 2: Echocardiographic in Table 3), which revealed that Rest-LASr, Rest-LAScd, Rest-LAVI-MAX, and Rest-*E*/*e*' were independent predictors of METS. Then, we combined clinical predictors (Age, BSA, and Target_HR) with resting ultrasound predictors (Rest-LASr, Rest-LAScd, Rest-LAVI-MAX, and Rest-*E*/*e*') and found that Rest-LASr, Rest-LASd, Rest-LAVI-MAX, and Rest-*E*/*e*' were still independent predictors (such as Model 3 in Table 3: combined clinical and echocardiographic predictors). Finally, we discovered that Rest-LASr, Rest-LAVI-MAX, and Rest-*E*/*e*' were independent resting echocardiographic predictors of METS (as Model 4 in Table 3), when we integrated the resting echocardiographic predictors with the clinical predictors (Age, BSA, and Target_HR).

**Table 3:** Multivariate regression models.

	B	SE	Lower	Upper	Stander_B	Pr(>|t|)
Model 1: clinical						
Age (year)	0.043	0.052	−0.058	0.145	0.581	0.402
Target_HR (beat/min)	0.103	0.046	0.013	0.193	1.54	0.027*
BMI (kg/m^2^)	−0.18	0.104	−0.383	0.024	−0.592	0.087
BSA (m^2^)	4.303	1.884	0.61	7.996	0.816	0.025*
Model 2: echocardiographic						
Model LASR						
Rest-LASR (%)	0.23	0.034	0.164	0.296	1.245	<0.001*
LVMI (g/m^2^)	0.0,002	0.002	−0.004	0.005	0.014	0.922
*E*/*e*'_Rest (m/s)	−0.04	0.023	−0.085	0.004	−0.258	0.076
Rest-LAVI (mL/m^2^)	−0.068	0.011	−0.089	−0.047	−1.193	<0.001*
Rest-LA_EF (%)	−0.008	0.015	−0.036	0.021	−0.077	0.609
Model LASCD						
Rest-LASCD (%)	−0.108	0.033	−0.173	−0.043	−0.546	0.002*
LVMI (g/m^2^)	−0.002	0.003	−0.008	0.003	−0.145	0.369
E/e'_Rest (m/s)	−0.068	0.026	−0.119	−0.018	−0.435	0.009*
Rest-LAVI (mL/m^2^)	−0.09	0.012	−0.113	−0.067	−1.569	<0.001*
Rest-LA_EF (%)	0.009	0.017	−0.024	0.042	0.095	0.585
Model LASCT						
Rest-LASCT (%)	−0.021	0.04	−0.099	0.058	−0.088	0.606
LVMI (g/m^2^)	−0.002	0.003	−0.008	0.004	−0.128	0.458
*E*/*e*'_Rest (m/s)	−0.078	0.027	−0.131	−0.025	−0.495	0.005*
Rest-LAVI (mL/m^2^)	−0.099	0.012	−0.123	−0.076	−1.731	<0.001*
Rest-LA_EF (%)	0.014	0.018	−0.021	0.049	0.141	0.444
Model 3: combined clinical and echocardiographic						
Model LASR						
Rest-LASR (%)	0.221	0.032	0.159	0.283	1.196	<0.001*
Age (year)	0.056	0.027	0.003	0.11	0.753	0.042*
Target_HR (beat/min)	0.064	0.025	0.016	0.113	0.961	0.011*
BSA (m^2^)	−0.27	0.702	−1.645	1.106	−0.051	0.702
*E*/*e*'_rest	−0.046	0.022	−0.089	−0.004	−0.294	0.036*
Rest-LAVI (mL/m^2^)	−0.063	0.01	−0.082	−0.044	−1.106	<0.001*
Model LASCD						
Rest-LASCD (%)	−0.103	0.032	−0.166	−0.04	−0.521	0.002*
Age (year)	0.054	0.032	−0.008	0.117	0.728	0.093
Target_HR (beat/min)	0.066	0.029	0.01	0.123	0.993	0.024*
BSA (m^2^)	0.328	0.815	−1.27	1.926	0.062	0.689
*E*/*e*'_rest	−0.069	0.025	−0.118	−0.02	−0.439	0.007*
Rest-LAVI (mL/m^2^)	−0.09	0.01	−0.11	−0.07	−1.571	<0.001*
Model LASCT						
Rest-LASCT (%)	−0.045	0.038	−0.12	0.03	−0.19	0.242
Age (year)	0.055	0.034	−0.011	0.121	0.732	0.106
Target_HR (beat/min)	0.072	0.03	0.013	0.132	1.083	0.019*
BSA (m^2^)	0.483	0.853	−1.188	2.155	0.092	0.572
*E*/*e*'_rest	−0.073	0.026	−0.124	−0.021	−0.463	0.007*
LAVI (mL/m^2^)	−0.097	0.01	−0.118	−0.076	−1.69	<0.001*
Model 4: ALL						
Rest-LASR (%)	0.229	0.039	0.152	0.306	1.237	<0.001*
Rest-LASCD (%)	0.011	0.034	−0.055	0.078	0.057	0.738
Age (year)	0.056	0.027	0.003	0.11	0.754	0.043*
Target_HR (beat/min)	0.065	0.025	0.016	0.113	0.964	0.011*
BSA (m^2^)	−0.275	0.706	−1.658	1.108	−0.052	0.698
E/e'_rest	−0.046	0.022	−0.089	−0.003	−0.292	0.038*
Rest-LAVI (mL/m^2^)	−0.063	0.01	−0.082	−0.044	−1.104	<0.001*

* *P* < 0.05.

### 3.4. Predictive ability

**Figure 4:**
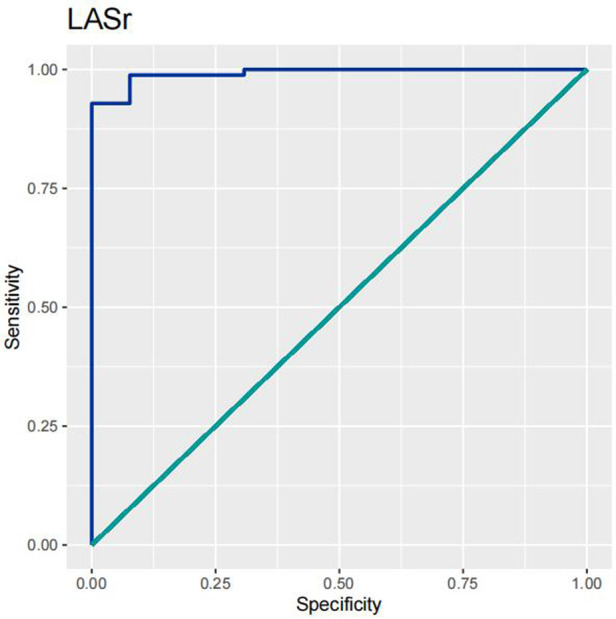
ROC curves of LASr.

**Figure 5:**
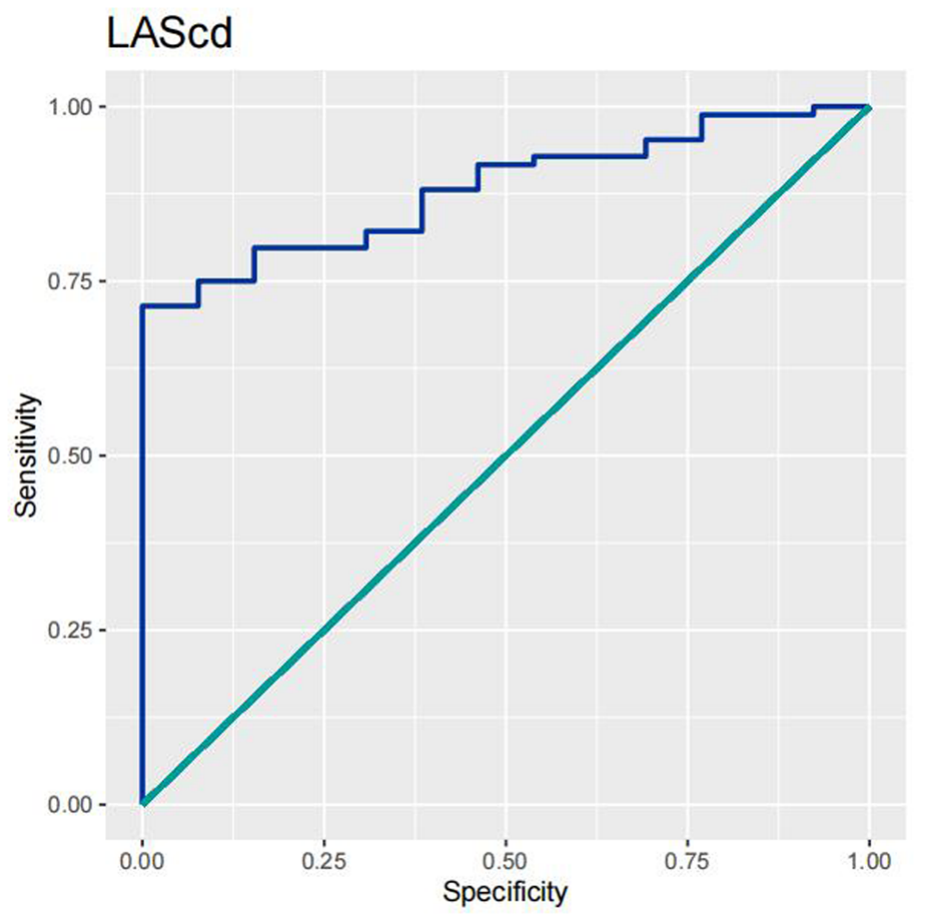
ROC curves of LAScd.

**Figure 6:**
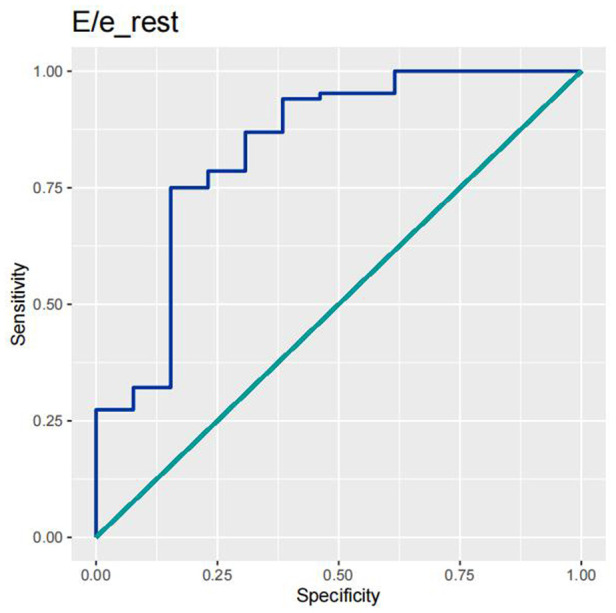
ROC curves of *E*/*e*'-rest.

**Figure 7:**
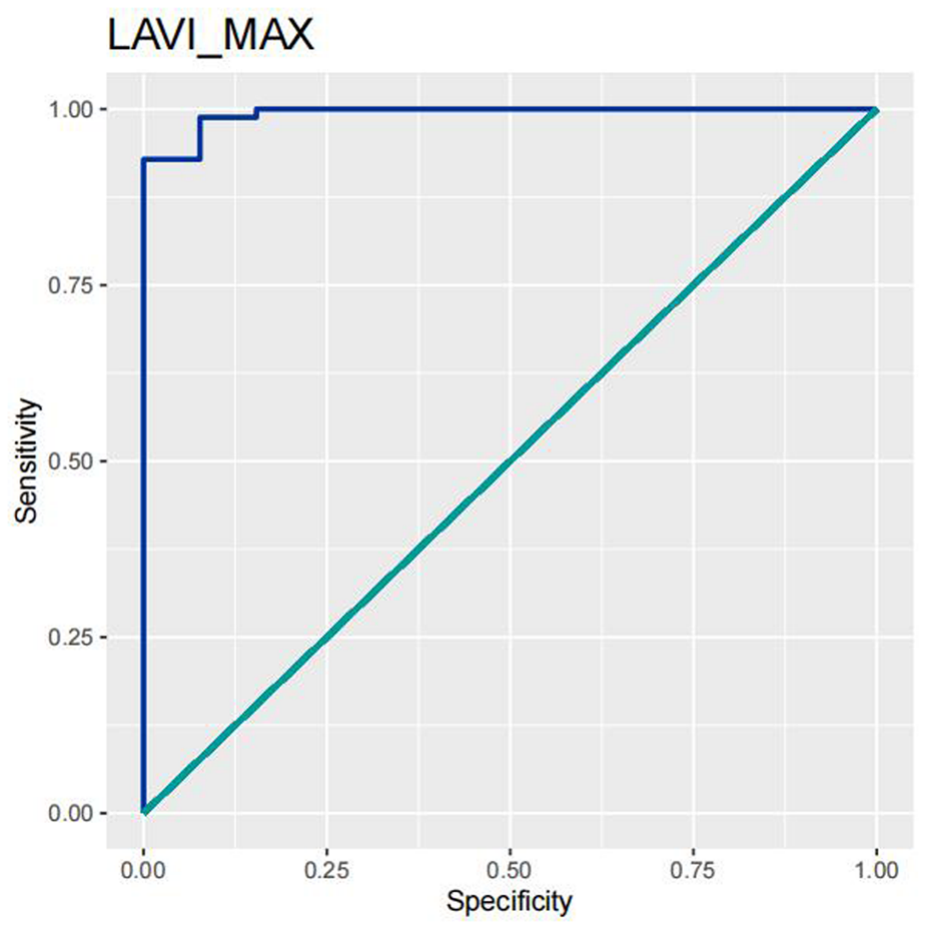
ROC curves of LAVI_MAX.

**Figure 8:**
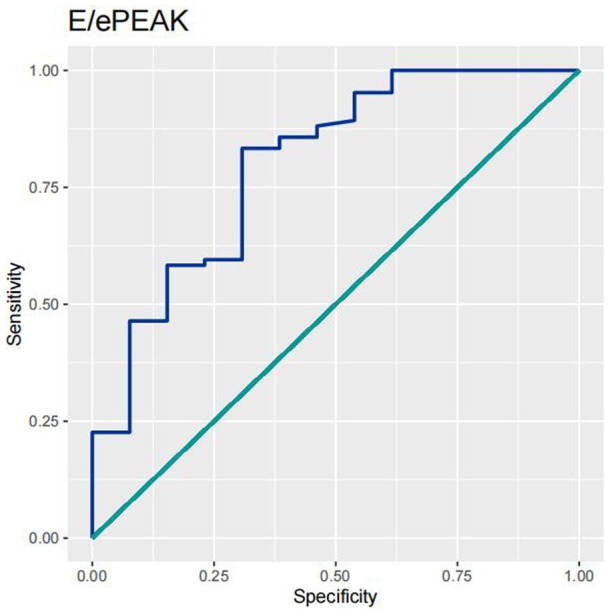
ROC curves of *E*/*e*'-PEAK.

**Figure 9:**
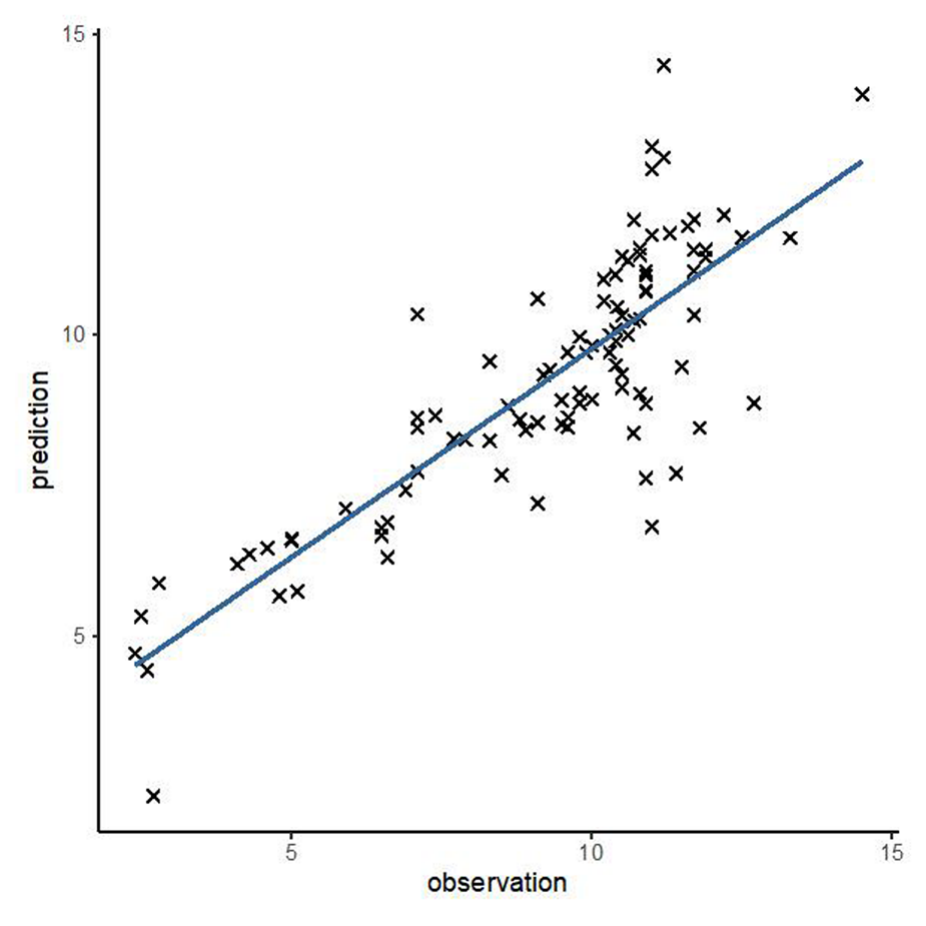
Overlay scatter plots of METS achieved vs. predicted METS.

In order to test the predictive ability of LASr, Rest-LAVI-MAX, and Rest-*E*/*e*', the ROC and AUC of the above parameters were compared. The LASr (AUC, 0.990; specificity, 1; sensitivity, 0.93; cutoff value 8%, *P* < 0.01) was the strongest resting echocardiographic predictor of decreased METS and showed better performance than *E*/*e*'-Peak (AUC, 0.753; specificity, 0.85, sensitivity 0.61, cutoff value 13.5) (as shown in Figures 4–8, Tables 4, 5). In order to evaluate the clinical value of LASr in predicting impairment in METS, we calculated the correlation coefficients and the corresponding *P*-values between clinical predictors (Age, Target_HR, BMI, and BSA) and plotted a multivariate linear regression model (METS:LASr + Age + Target_HR), and a strong positive linear relationship between the predicted METS of the model to METS achieved with exercise was observed (as shown in Figure 9). Therefore, in HCM patients, adding the LASr to age, Target_HR, and BSA can provide a robust model for predicting METS. In order to investigate whether LASr and LAVI were suitable for different subtypes of HCM, all HCM patients were divided into three groups (AHCM, asymmetric HCM, and obstructive HCM), and only Rest-LASr showed a statistical difference among the three groups. Rest-LASr showed a good performance in different subgroups (as shown in Table 6).

**Table 4:** The pairwise comparison of each index on the AUC.

Variable	AUC	Standard error	95% confidence interval	Significance (*P*-value)	Delong test pairwise comparison with *E*/*e*'-Peak (*P* value)	Delong test pairwise comparison with LASr (*P*-value)
Rest-LASr	0.990	0.006	0.978 to 1	*P* < 0.01	0.0,001	NA
Rest-LAScd	0.890	0.032	0.826 to 0.953	*P* < 0.01	0.0,701	0.0,016
*E*/*e*'_Rest	0.736	0.092	0.555 to 0.917	*P* < 0.01	0.8,422	0.0,068
Rest-LAVI_MAX	0.990	0.006	0.978 to 1	*P* < 0.01	0.0,001	0.9,520
*E*/*e*'-Peak	0.753	0.062	0.631 to 0.874	*P* < 0.01	NA	0.0,001

**Table 5:** Specificity and sensitivity of LASr, LASct, *E/e*'-rest, and *E/e*'-peak.

Variable	Threshold	95% confidence interval	Specificity	Sensitivity
Rest-LASR (%)	8	6.5 to 8	1.00	0.93
Rest-LASCD (%)	−4.5	−4.5 to −3.5	1.00	0.74
*E*/*e*'_Rest	18.48	11.71 to 30.5	0.62	0.86
Rest-LAVI (mL/m^2^)	53.5	51.5 to 59	1.00	0.92
*E/e*'-Peak	13.5	10.25 to 18.75	0.85	0.61

**Table 6:** Clinical, echocardiographic, and exercise characteristics in HCM.

	Overall (*N* = 97)	AHCM (*N* = 22)	Asymmetric HCM (*N* = 50)	Obstructive HCM (HOCM) (*N* = 25)	*P*
METS_index = 1 (%)	84 (86.6)	20 (90.9)	45 (90.0)	19 (76.0)	0.195
METS [mean (SD)]	9.25 (2.62)	9.60 (2.94)	9.29 (2.37)	8.86 (2.84)	0.63
Demographic and comorbidities					
Gender = M (%)	68 (70.1)	19 (86.4)	33 (66.0)	16 (64.0)	0.164
Age (year) [mean (SD)]	46 (13)	52 (13)	44 (13)	44 (12)	0.052
Target_HR (beat/min) [mean SD)]	172 (14)	167 (13)	173 (16)	173 (12)	0.224
Height (cm) [mean (SD)]	164.65 (8.26)	167.27 (8.17)	163.94 (7.40)	163.76 (9.73)	0.239
Weight (kg) [mean (SD)]	65.73 (11.97)	66.36 (9.02)	64.16 (11.11)	68.32 (15.40)	0.355
BSA (m^2^) [mean (SD)]	1.69 (0.19)	1.72 (0.15)	1.67 (0.17)	1.72 (0.25)	0.427
BMI (kg/m^2^) [mean (SD)]	24.15 (3.29)	23.70 (2.76)	23.81 (3.43)	25.23 (3.33)	0.162
Rest-SBP (mmHg) [mean (SD)]	125 (24)	123 (18)	125 (21)	126.82 (33)	0.868
Rest-DBP (mmHg) [mean (SD)]	77 (13)	76 (12)	78 (13)	75.96 (12)	0.748
Rest_HR (beat/min) [mean (SD)]	78 (11)	77 (10)	77 (10)	81 (13)	0.31
Ventricular arrhythmias (%)	10 (10.30)	1 (4.54)	4 (8.00)	5 (20.00)	0.164
Atrial arrhythmias (%)	4 (4.12)	2 (9.09)	1 (2.00)	1 (4.00)	0.378
Baseline echocardiography					
LVM I (mL/m^2^) [mean (SD)]	143.53 (58.63)	118.69 (69.01)	147.79 (47.52)	156.85 (64.68)	0.062
Rest-LVEF [mean (SD)]	0.73 (0.06)	0.72 (0.05)	0.72 (0.06)	0.74 (0.07)	0.272
Rest-LV-GLS (%) [mean (SD)]	−19.68 (3.84)	−18.89 (3.73)	−19.33 (4.03)	−21.08 (3.28)	0.095
Rest_E (m/s) [mean (SD)]	0.74 (0.23)	0.75 (0.16)	0.73 (0.22)	0.75 (0.29)	0.889
Rest_A (m/s) [mean (SD)]	0.73 (0.27)	0.73 (0.29)	0.70 (0.23)	0.81 (0.33)	0.252
Rest_E/A [mean (SD)]	1.16 (0.56)	1.18 (0.51)	1.16 (0.50)	1.13 (0.71)	0.949
Rest_e' (m/s) [mean (SD)]	0.06 (0.02)	0.07 (0.02)	0.06 (0.02)	0.06 (0.02)	0.453
Rest_a (m/s) [mean (SD)]	0.08 (0.02)	0.09 (0.02)	0.08 (0.02)	0.08 (0.03)	0.112
*E/e*'-rest [mean (SD)]	14.01 (6.36)	12.60 (5.50)	14.26 (6.28)	14.75 (7.25)	0.477
Diastolic grade					
Rest_LASr (%) [mean (SD)]	13.22 (5.41)	15.86 (6.47)	12.28 (4.52)	12.76 (5.51)	**0**.029*
Rest_LAScd (%) [mean (SD)]	−7.04 (5.04)	−8.95 (5.35)	−6.62 (4.37)	−6.20 (5.74)	0.121
Rest_LASct (%) [mean (SD)]	−5.20 (4.24)	−6.50 (3.04)	−5.12 (3.98)	−4.20 (5.38)	0.177
Rest_LAVI_MAX (mL/m^2^) [mean (SD)]	42.72 (17.43)	42.77 (24.33)	41.42 (13.67)	45.28 (17.48)	0.669
Rest_LA_EF (%) [mean (SD)]	37.62 (10.27)	38.36 (9.59)	37.32 (10.04)	37.56 (11.60)	0.925
Exercise parameters					
Peak-SBP (mmHg) [mean (SD)]	171 (33)	166 (25)	169 (35)	178 (35)	0.433
Peak-DBP (mmHg) [mean (SD)]	74 (17)	75 (13)	73 (19)	76 (17)	0.756
Peak-HR (beat/min) [mean (SD)]	157 (23)	158 (20)	157 (25)	157 (22)	0.991
*E/e'*-Peak [mean (SD)]	14.43 (6.38)	12.78 (4.40)	14.29 (6.21)	16.15 (7.83)	0.191
Peak-LVEF [mean (SD)]	0.83 (0.09)	0.85 (0.04)	0.83 (0.06)	0.83 (0.17)	0.758

* *P* < 0.05.

## 4. Discussion

This study assessed the relationship between LAS and METS in HCM patients using treadmill stress echocardiography combined with 3D-STI. METS, Rest-LASr, Rest-LAScd, and Rest-LASct in HCM patients were significantly lower than those in the normal group. There were significant differences in age, Target_HR, LVMI, Rest-LAVI-MAX, *E*/*e*'-Rest, *E*/*e*'-Peak, Rest-LASr, Rest-LAScd, and Rest-LASct between the groups with METS > 6.0 and ≤ 6.0. Rest-LASr had the strongest correlation with METS ≤ 6.0, which was an independent resting echocardiographic predictor of METS. This parameter also yielded good results in different subgroups (AHCM, asymmetric HCM, and obstructive HCM). Compared with the traditional parameters (Peak-*E*/*e*' and Rest-*E*/*e*'), Rest-LASr was better at predicting METS ≤ 6.0. Furthermore, a robust multivariate model (LASr + Age + Target_HR) was constructed for METS prediction.

HCM is an autosomal dominant genetic disease. At present, only 40%–60% of HCM is caused by sarcomere gene mutation, and the rate of incidence of HCM caused by unknown gene mutation and non-gene mutation accounts for approximately 5%–10%. The cause and mechanism of HCM remain unidentified in 25%–30% of patients (1), and previous research examined at least 10 out of 1,500 mutations associated with HCM, which can pose significant challenges and confusion for physicians and patients during the clinical diagnosis and treatment process. This can be seen from the updated diagnostic criteria for HCM in the 2020 AHA/ACC guidelines (8): the hypertrophy of any segment of the myocardium or special part of the ventricular wall ≥1.5 cm or ≥1.3 cm with a positive family history or a positive genetic test. Gene testing has been included in the guidelines, which needs to be combined with other imaging methods. Following a comprehensive analysis, this study took into account the fact that relying solely on genetic testing as the inclusion criteria for HCM patients may result in the exclusion of individuals with non-genetic mutations or unknown etiology. Therefore, the HCM patients included in this study were diagnosed by the Department of Cardiology of Sichuan Provincial People's Hospital. The clinical diagnostic criteria were also based on the corresponding guidelines (6–8), the diagnostic and inclusion criteria for HCM, to encompass a wide range of patients with different types and phenotypes of the condition. This approach aims to assist physicians in optimizing the clinical diagnosis and treatment plan of HCM.

The LAS was measured using 3D-STI to assess the change in length of the entire atrial myocardium in the tangential direction. The end-diastolic period of the ventricle was used as the zero baseline of the LAS curve to generate the longitudinal strain from each atrial segment. The LAS includes LASr, LAScd, and LASct, which reflect the functions of the left atrium in three different phases, namely, reservoir function, conduit function, and contraction function. The LASr corresponds to left ventricular isovolumic contraction and isovolumic relaxation, and the left ventricle coordinates the dilation of the left atrium through the motion of the mitral annulus. The pulmonary veins supply blood to the left atrium, causing the atrial wall to stretch to its peak state just before the opening of the mitral valve, which reflects the relaxation of the left ventricle. The LAScd corresponds to the early diastolic period. When the left atrium empties and blood flows into the left ventricle through the opened mitral valve, the left ventricle is filled, leading to a decrease in the LAScd (the strain curve is negative). The LAScd is regulated by atrial compliance and left ventricular relaxation. The LASct corresponds to the late diastole period characterized by left atrial contraction and pump function, which depends on the venous returning and end-diastolic pressure (19, 20). Compared with the tissue Doppler, the LAS is less dependent on angle and load, which can distinguish the active and passive motions of myocardial tissue, and evaluate the left atrial function more objectively and sensitively. The LAS has been shown to have better repeatability and feasibility (21). As shown in Table 1, the resting LASr, LAScd, and LASct of the normal group patients (44.40 ± 8.20%, −27.98 ± 6.45%, −16.41 ± 3.74%) were significantly higher than those of the HCM group patients (13.22 ± 5.41%, −7.04 ± 5.04%, −5.20 ± 4.24%) (*P* < 0.05).

Keles et al. (22) found that patients who had premature ventricular contraction showed a significant decrease in the LAS compared with those in the control group. Ventricular arrhythmias can lead to atrial and ventricular remodeling, and 4D-LAQ can quantitatively detect these changes at an early stage. Similar results were found in this study, as shown in Table 2. The Rest-LASr, Rest-LAScd, Rest-LASct, and Rest-LA-EF (4.62 ± 1.56%, −2.23 ± 1.17%, −1.69 ± 3.37%, and 27.00 ± 8.69%, respectively) were dramatically reduced in the HCM-1 group, and the incidence of ventricular arrhythmias was significantly higher (*P* < 0.01). Moreover, the conventional ultrasound parameters Rest-e decreased slightly; *E*/*e*'_rest and Rest_GLS hovered at the edge of the normal range; and only Rest_LAVI showed a significant increase. Classifying diastolic function in HCM patients using the previous method of diastolic dysfunction assessment would result in numerous uncertain factors. However, it is worth noting that the LAS can sensitively reflect diastolic dysfunction. Previous studies (23–25) also found that left atrial dysfunction may predate the abnormality of LAVI. An abnormal hypertrophy of cardiomyocytes, interstitial hyperplasia, and fibrosis in HCM patients can cause a thickening of the ventricular wall, a decrease in compliance, an increase in left ventricular end-diastolic pressure, and obstructed left ventricular blood filling. Varying degrees of congestion may occur in the left atrium and lungs, leading to a further reduction in the compliance of the left atrial wall, pulmonary blood vessels, and pulmonary interstitial structures. This, in turn, can cause an increase in pulmonary vascular pressure, affecting the return of blood flow to the left atrium during exercise, and ultimately resulting in a significant decrease in the LASr, LAScd, and LASct. Cauwenberghs et al. (26) demonstrated that when LASr was <23%, subclinical LA dysfunction was associated with an increased risk of future adverse cardiac events. Several studies (27–30) have shown that the LAS tends to progressively progress in all stages of diastolic dysfunction, and LASr can differentiate diastolic dysfunction more accurately and sensitively. Kurt et al. (31) showed that the LASct was significantly correlated with left ventricular end-diastolic pressure and B-type natriuretic peptide levels. The LASr is closely associated with pulmonary capillary wedge pressure and early treatment response, making it a sensitive marker of left atrial fibrosis and a strong prognostic indicator (32). Therefore, combining LAS measurement and left atrial function assessment can provide a basis for diagnosing and classifying subclinical left atrial dysfunction, predicting new-onset atrial fibrillation, and identifying adverse cardiovascular events in clinical practice (33).

As can be seen from Table 1, there was no significant difference in atrial arrhythmias between the METS ≤ 6.0 and the METS > 6.0 groups of patients. Similarly, there was still no significant difference in atrial arrhythmias among the AHCM, asymmetric HCM, and obstructive HCM groups (Table 6). Atrial arrhythmias refer to any type of abnormal heart rhythm originating from the atria of the heart. The common clinical manifestations are atrial premature beats, atrial tachycardia, atrial flutter, and atrial fibrillation. The causes of atrial arrhythmias include myocardial ischemia, electrolyte disturbances, and thyroid dysfunction. The main risks of atrial arrhythmias involve alterations in vital signs, such as heart rate and blood pressure, as well as the possibility of thromboembolic events. The onset of atrial arrhythmias in HCM patients is associated with an increased volume of the left atrium and left ventricle, as well as elevated left ventricular end-diastolic pressure. To some extent, arrhythmia may be induced when the volume of the left atrium and left ventricle, as well as the left ventricular end-diastolic pressure, reach a certain threshold. A previous study (34) showed that asymptomatic cats with HCM had similar numbers of atrial and ventricular premature beats compared with the control cats. In this study, there was no significant difference in atrial premature beats between the METS ≤ 6.0 and the METS > 6.0 groups (Table 2). Similarly, as shown in Table 6, there was no significant difference in atria premature beats among the three subtypes of HCM, whereas the LASr was significantly decreased in these groups. Therefore, we speculate that the LASr is more sensitive in reflecting the changes in the atrial structure and function than atrial arrhythmia, which can better predict abnormal atrial function at an early stage. Nevertheless, premature atrial contraction alone is insufficient for the early detection of pathological changes in HCM patients.

Impairment of METS is a multidimensional mechanism that includes physical decline secondary to aging, skeletal muscle bioenergetics, respiratory mechanics, and cardiovascular dysfunction (35). Pandey et al. (36, 37) showed that for individuals with normal exercise function, the METS gradually declined with age, mainly due to a reduction in cardiac reserve. Rowin et al. (38) found that METS can be used for the individualized quantitative evaluation of HCM patients who are undergoing radiofrequency ablation, myocardial resection, or heart transplantation. This is significant for determining treatment options and stratifying risk. The diastole of the left ventricle and the function of the left atrium have an important impact on exercise tolerance, especially the left atrium, which can be affected by both preload (left ventricular diastole) and afterload (pulmonary blood flow). Von Roeder et al. (39) and Kusunose et al. (40) found that a decrease in METS was associated with left ventricular diastolic dysfunction, as well as the size and function of the left atrium. In addition, the LASr can explain the relationship between cardiac and peripheral exercise capacity reduction (41), whereas the association between METS and left ventricular systolic function is relatively weak. Patel et al. (42) showed that diminished exercise capacity was associated with inadequate biatrial function, insufficient left atrial functional reserve, and left ventricular diastolic dysfunction. In addition, Rest-LASr was found to be independently related to decreased METS, whereas LV-GLS did not show a correlation with METS. This study revealed that MET in the HCM group was significantly reduced, and the HCM-1 subgroup had an even more significant decrease in METS. Moreover, the resting LASr, LAScd, and LASct were significantly lower in the HCM-1 group. METS had the strongest positive correlation with Rest-LASr (*r* = 0.820; *P* < 0.01), while it was negatively correlated with Rest-LAScd (*r* = −0.624; *P* < 0.01) and age (r = −0.495; *P* < 0.01). However, this study did not observe a significant difference in LV-GLS between the groups. Therefore, it can be inferred that in the HCM group, the reduced diastolic function of the left ventricle results in decreased blood returning to the left atrium from both the systemic and the pulmonary veins during exercise, leading to a significant reduction in longitudinal stretching and deformation of the left atrium, particularly in LASr and LASct. Such congestion in the left atrium and lungs could affect alveolar gas exchange during exercise, thereby causing obvious symptoms such as dyspnea, fatigue, chest tightness, and shortness of breath during exercise, and ultimately leading to a reduction in METS. LV-GLS primarily reflects ventricular systolic function, but it is not a reliable predictor of left atrial pressure and exercise tolerance. In clinical practice, it has been observed that a significant proportion of HCM patients do not exhibit any symptoms at rest, but only experience symptoms of increased filling pressure during exercise. Furthermore, their *E*/*e*' ratio may fall within the critical range or even appear normal at rest. Although both the 2016 ESC Heart Failure guidelines (43) and the 2016 American Society of Echocardiography (ASE)/European Association of Cardiovascular Imaging (EACVI) (44) have suggested that the *E*/*e*' ratio can be used to reflect the elevated LV filling pressure, which has been validated by invasive measurements (45), it is important to note that many factors can impact METS. The state of cardiopulmonary function is just one component among many. Telles et al. (46) showed that the LASr and LASct were independently associated with exercise pulmonary capillary wedge pressure, and the LASr was a dual reflection of left ventricular filling pressure and pulmonary blood flow. The primary objective of this study was to assess the direct relationship between METS and LASr, providing a more direct and clear perspective.

In addition, this study found that METS had the strongest correlation with Rest-LASr (*r* = 0.820), and its correlation with *E*/*e*'-Rest (*r* = −0.241; *P* < 0.01) was relatively weak. The ROC and AUC indicated that the LASr could predict MTES ≤ 6.0 with the largest AUC, with the highest sensitivity and specificity and the corresponding cutoff value of less than 8% (Figures 4–8, Tables 4, 5). This implies that the LASr is the most significant resting echocardiographic predictor of reduced METS. Rest-LASr and Rest-LAVI have better diagnostic performance than *E*/*e*'-rest, *E*/*e*'-Peak, and Rest-LAScd. Moreover, both LASr and LAVI had similar sensitivity, specificity, and AUC between the METS ≤ 6.0 and the METS > 6.0 groups, and there was no significant difference in the Delong test (Table 5). However, when the above two parameters were applied in different types of HCM, only LASr showed significant differences (Table 6). This, in turn, confirms the scientific hypothesis of this study and suggests that the methods utilized in this research can be implemented in clinical practice. We validated the reason behind exploring left atrial volume measurements, which included fully automatic measurements as well as those with additional user input such as adjusting alignment and mesh edits, by comparing them with the results from the “Triplane volume” tool, and found that the LAV was significantly impacted by load. Badano et al. (47) and Steele et al. (48) showed that the changes in the LAS occur prior to the changes in the left atrial volume and may even exist independently without any changes in the volume. On the other hand, the LAS was measured by using 3D-STI, where the strain calculation is based on the change in the length of different lines along each anatomical direction. To calculate the longitudinal strain, we sampled eight longitudinal lines, each connecting two opposite LA basal points, from an automatically constructed triangular mesh (Figure 2). This allowed us to measure tangential changes in the length of the entire atrial myocardia without being affected by valvular disease, heart failure, arrhythmia, image acquisition section, etc. Hence, the LASr can be accurately measured even when combined with related diseases, which are more common in clinical practice. In addition, the LASr is independent of LAV, LV-GLS, age, LVEF, and *E*/*e*' (49), and the LAS is less load-dependent than LAVI. Furthermore, Rest-LASr can be easily obtained in the resting state, which is more clinically applicable. Therefore, the LASr can be an accurate method to reflect left ventricular diastolic function and predict METS with high sensitivity and specificity in situations where common echocardiographic parameters may not be able to rapidly determine diastolic dysfunction, when HCM patients are unable to undergo exercise testing because of various conditions (such as physical disability or severe obstruction or systemic disease affecting movement), or when there is an urgent clinical need to predict METS for treatment decisions.

## 5. Conclusion

The LASr has the strongest association with METS ≤ 6.0. The LASr is an independent resting predictor of METS ≤ 6.0, which displays good performance in different subtypes of HCM. Compared with the traditional parameters, Peak-*E*/*e*' and Rest-*E*/*e*', Rest-LASr is the best predictor. Rest-LASr can serve as a reliable method for HCM patients who are unable to undergo exercise testing but require an urgent evaluation of METS, which provides a basis for clinical treatment decision-making and treatment effect evaluation.

## 6. Limitations

This study also has some limitations. The first limitation is that it is a single-center study. Moreover, this study did not further refine the analysis for simultaneous factors that affect METS, such as systemic inflammation, endothelial dysfunction, changes in the intracellular and extracellular structures of cardiomyocytes, skeletal muscle bioenergetics, pulmonary functional status, mitral and tricuspid regurgitation, and left ventricular outflow tract obstruction. The measurement results of the LAS may be affected by the different algorithms used in the analysis software of various ultrasonic diagnostic instruments. It may be necessary to further expand the sample size and include a wider range of ultrasonic diagnostic instrument models and analysis software to accurately analyze the LAS and METS in HCM patients. In addition, follow-up observations should be conducted to assess the occurrence of cardiovascular events in HCM patients with METS below 6.0. Chung et al. (50) showed that sarcomere gene mutations were associated with left atrial dysfunction in HCM, which was independent of LV filling pressure and LAVI. The findings suggested a relationship between the genes and the structure and function of HCM. In our study, due to various factors such as duration, cost, and family-related issues, many HCM patients did not undergo genetic testing. Therefore, HCM patients could not be grouped according to the genetic results in order to uncover the relationships among some specific genes, mechanics, cardiac work, structure, and function, which is also one of the limitations of this study. These are also aspects that we hope to improve in the follow-up research.

## Data Availability

The raw data supporting the conclusions of this article will be made available by the authors without undue reservation.
